# The Role of Melanotransferrin (CD228) in the regulation of the differentiation of Human Bone Marrow-Derived Mesenchymal Stem Cells (hBM-MSC)

**DOI:** 10.7150/ijms.53650

**Published:** 2021-02-04

**Authors:** Maria Dubon, Sooho Lee, Ji-Hong Park, Jae-Yong Lee, Dongchul Kang

**Affiliations:** 1 Ilsong Institute of Life Science, Hallym University, Anyang, Gyeonggi-do 14066, Republic of Korea.; 2 Department of Biomedical Gerontology, Graduate School of Hallym University, Chuncheon, Gangwon-do 24252, Republic of Korea.; 3 Department of Biochemistry, College of Medicine, Hallym University, Chuncheon, Gangwon-do 24252, Republic of Korea.

**Keywords:** Melanotransferrin, differentiation, osteogenesis, adipogenesis, mesenchymal stem cells, cell surface markers

## Abstract

Melanotransferrin (CD228), firstly reported as a melanoma-associated antigen, is a membrane-bound glycoprotein of an iron-binding transferrin homolog. CD228 was found to be expressed significantly higher in human bone marrow-derived mesenchymal stem cells (hBM-MSC) than in human embryonic fibroblasts (FB) by RT-PCR, western blotting and flow cytometry. The expression of CD228 declined in aged hBM-MSC as osteogenesis-related genes did. We examined a possible role for CD228 in the regulation of osteogenesis and adipogenesis of hBM-MSC. Surprisingly, siRNA-mediated CD228 knockdown increased the expression of the transcription factor DLX5 and enhanced osteogenesis of hBM-MSC evidenced by an increased expression of the runt-related transcription factor 2 (RUNX2), osterix (Osx), and osteocalcin (OC), as well as higher alkaline phosphatase (ALP) activity and extracellular calcium deposition. Interestingly, hBM-MSC transfected with CD228 siRNA also showed an increase in intracellular lipid level during adipogenesis, indicated by oil red O staining of differentiated adipocytes. Overall, our study unveils CD228 as a cell surface molecule expressed by young hBM-MSC, but not by FB. It also provides evidence to suggest a role for CD228 as a negative regulator of osteogenesis and of lipid accumulation during adipogenesis in hBM-MSC *in vitro.*

## Introduction

Mesenchymal stem cells (MSC) are adult stem cells that possess the ability to self-renew and to differentiate into multiple tissue types 1-3. At first, they were thought to differentiate into mesenchymal tissues such as bone, fat and cartilage only; however, as their use in therapeutic settings grew in popularity, various studies have reported that MSC are able to transdifferentiate into specialized cells of other tissues such as nerve cells, cardiomyocytes, and several skin cell types, among others 4-12. Their relatively easy accessibility, lack of ethical issues, and therapeutic potentials in immunomodulation, anti-inflammation and tissue regeneration, make MSC one of the most suitable sources for the development of stem cell-based therapies 13-16.

Initially described in the bone marrow, MSC have been isolated from a wide array of tissues including adipose tissue, umbilical cord, and placenta 17. MSC share several characteristics with fibroblastic cells (FB), which are found residing in close contact with MSC in tissues, including the bone marrow 18, 19. When isolated and cultured *in vitro*, both cell types present plastic-adherent growth, fibroblast-like morphology, and similar immunophenotypes 20-22. According to the consensus definition by the International Society for Cellular Therapy (ISCT), MSC are immunophenotypically defined by the expression of the cell surface markers CD73, CD90 and CD105 and their lack of CD34, CD45, CD14 or CD11b, CD19 or CD79α, and HLA-DR 23. However, as evidenced by numerous studies, FB from various sources could also be defined as MSC under this criteria, since FB also have been found to express the surface markers CD73, CD90, and CD105 20, 24, 25. Moreover, the more recently found capacity of FB to differentiate into mesenchymal tissues and to modulate the immune response, further hinders the ability to distinguish and isolate MSC from FB for their use in stem cell-based therapies 26, 27.

Several groups have addressed this issue by searching new cell surface markers that are specific to MSC but not to FB. Studies comparing the expression of cell surface molecules of FB and MSC have proposed novel cell surface markers such as CD10 and CD26 (expressed in FB but not in MSC), and CD106, and CD146 (expressed in MSC but not in FB) which could be used to discriminate these two cell populations 28-30. However, most of these cell surface markers were reported to vary according to factors such as the source of the cells used for the analysis (MSC and FB, both), among others 31. Furthermore, a study reported that the expression of certain cell surface markers, believed to characterize MSC such as CD106, decrease with aging in MSC *in vitro*, which further complicates their use in the characterization of MSC when they are passaged for expansion 32.

MSC-specific surface markers could also be involved in the regulation of the differentiation potential of MSC. Overexpression of the cell surface protein CD200 (a proposed novel MSC-specific marker) improved the differentiation potential and the immunoregulatory functions of MSC 33, 34. In addition, another study suggested that CD200-positive MSC had elevated osteogenic differentiation potential 35. It was also reported that CD271(+) MSC showed higher tri-lineage differentiation potential than their CD271(-) counterparts 36. These findings suggest that more than one cell surface markers could be involved in the regulation of, among other cellular processes, the differentiation of MSC.

Melanotransferrin (MTf), also known as CD228, is an iron-binding transferrin homolog membrane-bound glycoprotein. Firstly identified as a human tumor-associated antigen, it was later found to be expressed in normal tissues such as the salivary glands, eosinophils, brain capillaries, and cartilage 37-41. It is believed to play a role in iron metabolism, proliferation and migration in the tissues where it is expressed 41-44. Importantly, a study reported that CD228 is involved in the chondrogenic differentiation of mouse prechondrogenic ATDC5 cells and mouse pluripotent mesenchymal C3H10T1/2 cells *in vitro* 45. Expression of CD228 was found higher in MSC than in FB in a preliminary microarray-based comparative transcriptome analysis between FB and hBM-MSC (data not shown). Herein, we compared the expression of CD228 between hBM-MSC and FB, and investigated whether CD228 could be involved in the regulation of the differentiation of hBM-MSC towards the osteogenic and/or adipogenic lineage.

## Results

### CD228 was expressed in hBM-MSC but not in FB

**Figure 1:**
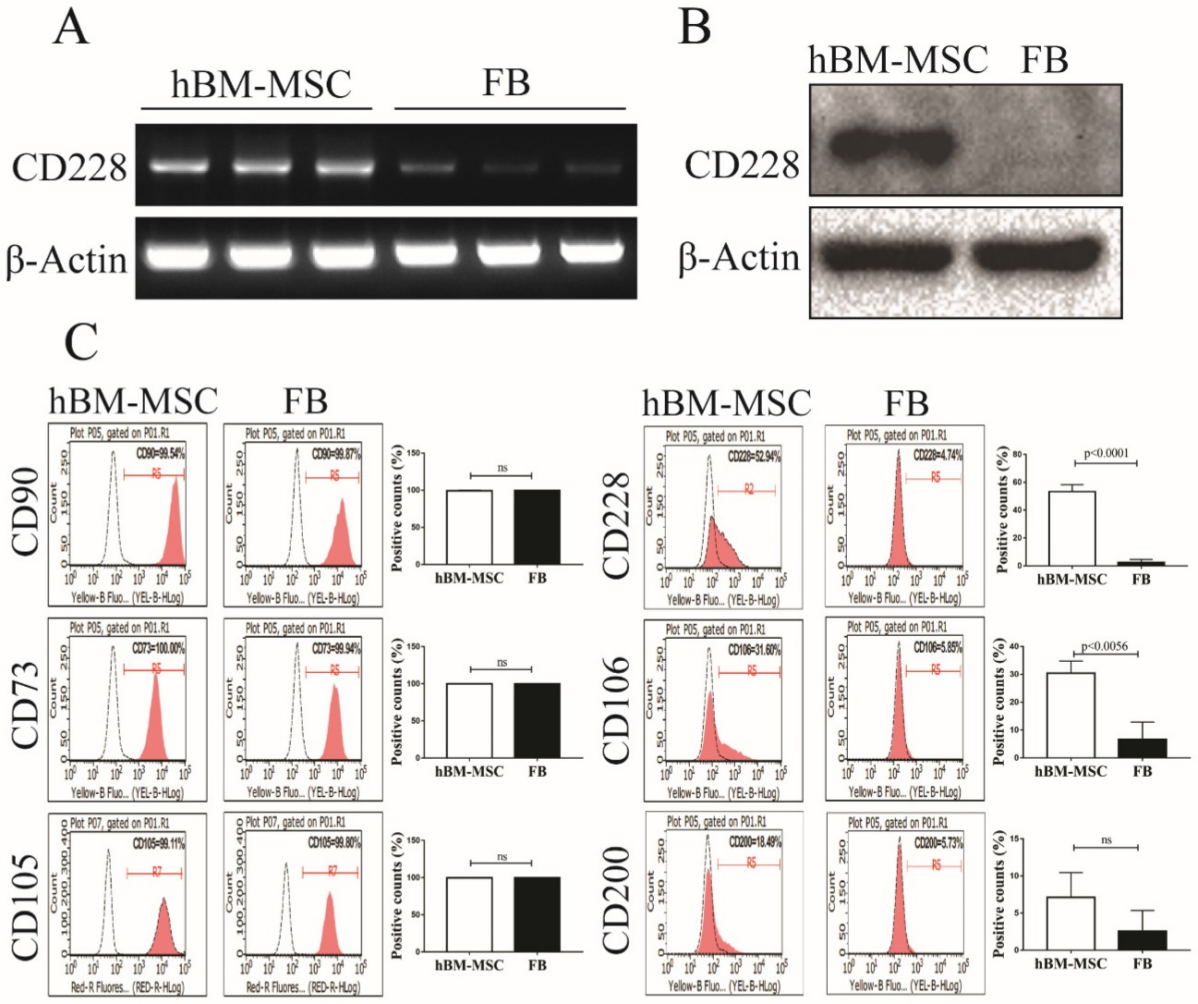
** Analysis of CD228 expression in hBM-MSC and FB.** Comparison of the expression of CD228 between hBM-MSC and human FB by (A) RT-PCR analysis, (B) western blot analysis, and (C) FACS analysis. Established MSC markers were also detected by FACS analysis in hBM-MSC and FB. β-Actin was used as an internal control for RT-PCR analysis and western blot analysis. Representative histograms show the expression of CD228 as indicated. Bar graphs show the percentage of positive events presented as the mean ± SD of three independent experiments. Red curves: positive samples, white curves: negative isotype control. p-values were calculated using the Student's t-test, ns: not significant.

Comparative microarray analysis between hBM-MSC and human FB in a preliminary study pointed us to a cell surface molecule CD228 that appeared to express higher in hBM-MSC than in FB. CD228 expression between hBM-MSC and FB was further compared by RT-PCR, western blotting and flow cytometry. RT-PCR and western blot analysis showed that CD228 expressed consistently and significantly higher in hBM-MSC compared to FB (Figure 1A and 1B). We next confirmed the expression of CD228 on hBM-MSC, but not on FB by flow cytometry, which showed an obvious positive shift of CD228 fluorescence signal in hBM-MSC over isotype control, but not in FB (Figure 1C). In contrast, both hBM-MSC and FB expressed the positive markers established by the ISCT (CD90, CD73, and CD105) for the characterization of MSC (Figure 1C). The identity of hBM-MSC was further confirmed by the positive expression of the MSC-specific cells surface markers CD106 and CD200 33, 46. As expected, FB did not express CD106 or CD200 (Figure 1C). These results clearly demonstrate CD228 as a potential hBM-MSC marker that distinguishes hBM-MSC from FB. Therefore, in this study we focused on further analyzing the expression and function of CD228 in hBM-MSC.

### CD228 expression decreased with aging in hBM-MSC

**Figure 2:**
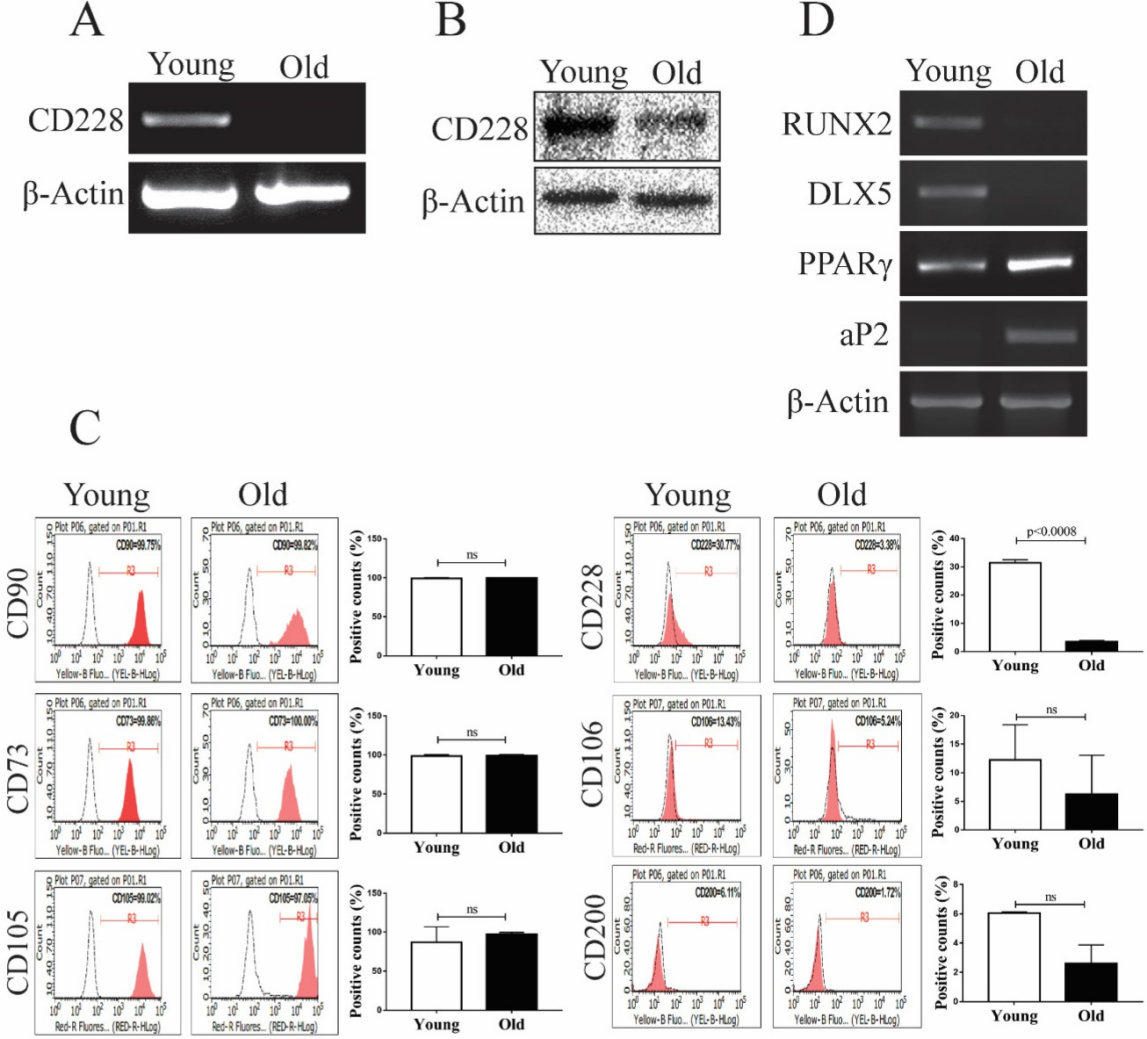
** Analysis of differentiation-related markers and CD228 between young and old hBM-MSC.** The expression of CD228 was analyzed in young and old hBM-MSC by (A) RT-PCR analysis, (B) western blot analysis, and (C) FACS analysis. Established MSC markers were also detected by FACS analysis in young and old hBM-MSC. β-Actin was used as an internal control. Representative histograms show the expression of CD228 as indicated. Bar graphs show the percentage of positive events presented as the mean ± SD of three independent experiments. Red curves: positive samples, white curves: negative isotype control. p-values were calculated using the Student's t-test, ns: not significant. (D) RT-PCR analysis for osteogenic or adipogenic differentiation-related genes in young and old hBM-MSC. β-Actin was used as an internal control.

The aging process changes the gene expression of hBM-MSC, as well as their intrinsic characteristics such as stemness, proliferation, and differentiation potential 47, 48. These changes can greatly influence the therapeutic and regenerative properties of hBM-MSC 49-51. We analyzed the effect of aging on the expression of CD228 using young (passages 4 to 6) and old (passages 16 to 18) hBM-MSC. Interestingly, we found that aging decreased CD228 mRNA and protein levels examined by RT-PCR and western blot analysis, respectively (Figure 2A and 2B). The decreased expression of CD228 on the surface of old hBM-MSC compared to young hBM-MSC was also confirmed by flow cytometry (Figure 2C). Aging also caused a slight decrease in the expression of the MSC-specific markers CD106 and CD200 (Figure 2C). In contrast, there was no change in the expression level of the established MSC markers CD90, CD73, and CD105 in old hBM-MSC compared to young hBM-MSC (Figure 2C). Studies have reported that the process of aging alters the differentiation potential of hBM-MSC, favoring adipogenesis while compromising osteogenesis 52, 53. Therefore, we compared the expression of differentiation-related genes between young and old hBM-MSC by RT-PCR (Figure 2D). We observed a decrease in the mRNA expression level of the well-known osteogenesis-related factor DLX5 54, 55. Furthermore, the expression level of the osteogenesis marker RUNX2 decreased in old hBM-MSC compared to young hBM-MSC (Figure 2D) 56. Conversely, old hBM-MSC showed an increase in the mRNA levels of the adipogenesis markers PPARγ and aP2 compared to young hBM-MSC (Figure 2D) 57. These findings, showing a decreased expression of the cell surface marker CD228 and osteogenesis-related genes with aging of hBM-MSC, led us to ask whether CD228 could also play a role in the lineage commitment/differentiation potential of hBM-MSC. Hence, we sought to determine whether CD228 was involved in the differentiation of hBM-MSC towards the osteogenic and/or adipogenic lineage *in vitro*.

### CD228 knockdown increased the expression of osteogenic differentiation-related genes in hBM-MSC

**Figure 3:**
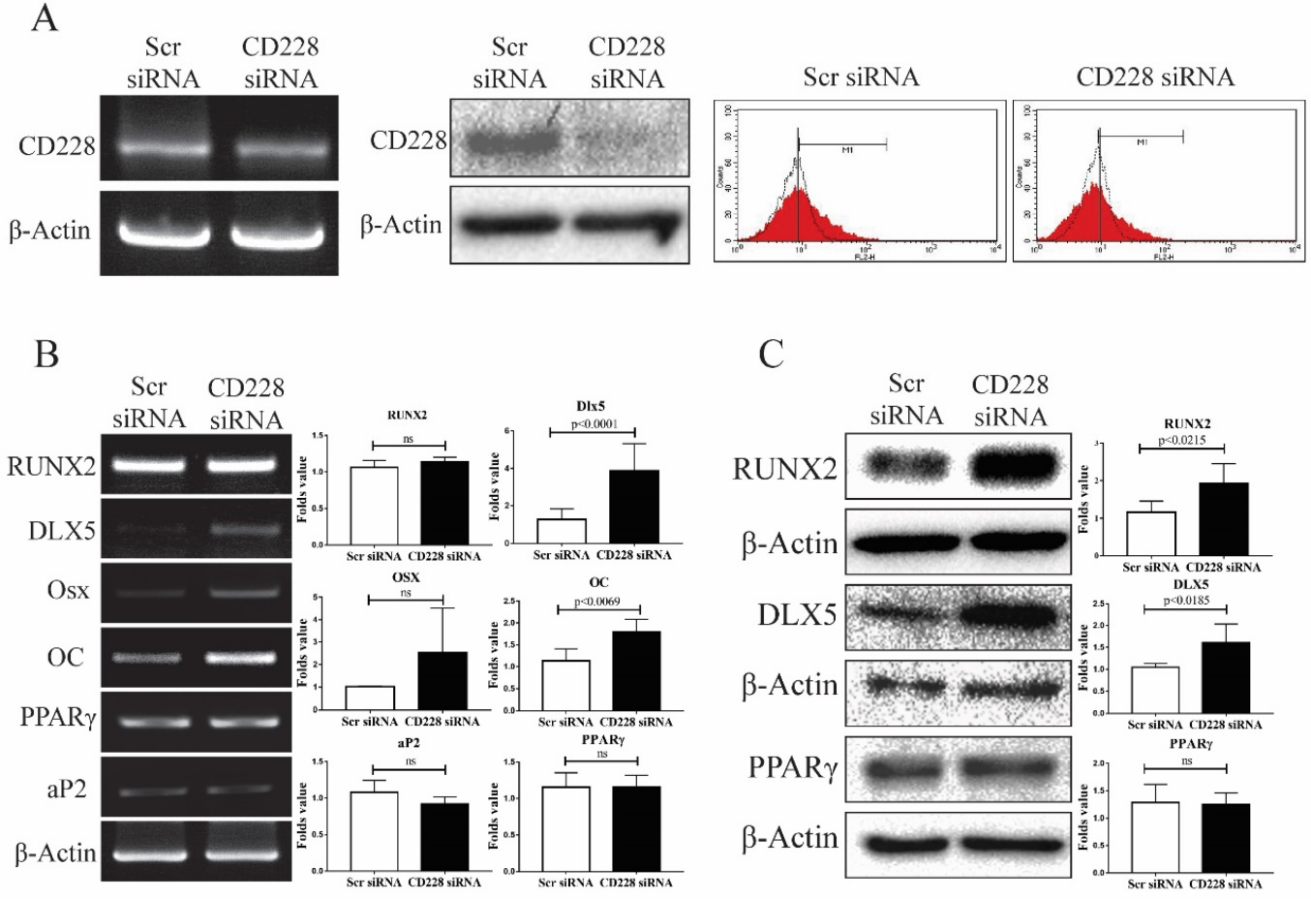
** Expression of osteogenesis or adipogenesis-related markers after CD228 knockdown in hBM-MSC.** hBM-MSC were transfected with scrambled siRNA (Scr siRNA) or siRNA against CD228 (CD228 siRNA) for 48 h. (A) Knockdown of CD228 by siRNA transfection was evaluated by RT-PCR (left), western blot analysis (middle), and FACS analysis (right). Red curves: positive samples, white curves: negative isotype control. β-Actin was used as an internal control. (B) RT-PCR analysis and (C) western blot analysis for the indicated osteogenesis- or adipogenesis-related markers in hBM-MSC transfected with Scr siRNA or CD228 siRNA for 48 h. β-Actin was used as an internal control. Bar graphs show the fold change in the expression of each gene for CD228 siRNA-transfected cells compared to Scr siRNA control ones. Data is presented as the mean ± SD of three to four independent experiments. p-values were calculated using the Student's t-test; ns: not significant.

To determine whether CD228 plays a role in the differentiation potential of hBM-MSC towards the osteogenic and/or the adipogenic lineages, we knocked down the expression of CD228 by transfection of siRNA and compared the expression of several differentiation-related genes between the control (scrambled siRNA) and CD228-knocked down hBM-MSC (CD228 siRNA). Compared to control hBM-MSC, transfection with CD228 siRNA decreased CD228 mRNA and protein levels, as well as its expression on the cell surface of hBM-MSC (Figure 3A left, middle, and right, respectively). To our surprise, siRNA-mediated CD228 knockdown increased mRNA and protein level of the transcription factor DLX5, a master regulator of osteogenesis 54, 55. Knockdown of CD228 in hBM-MSC increased the mRNA level of the osteoblast-specific gene osteocalcin (OC) and the protein level of RUNX2, an early transcription factor essential for osteoblast differentiation (Figure 3B and 3C) 56. Although not statistically significant, CD228 knockdown caused a slight increase in osterix (Osx), a late osteoblast-specific transcription factor (Figure 3B) 56. On the other hand, knockdown of CD228 did not cause a significant change in the expression level of the adipogenesis-related genes PPARγ and aP2 (Figure 3B and 3C) 57. Thus, these data suggest that the expression of CD228 might, to some degree, be involved in suppression of the osteogenic differentiation of hBM-MSC.

### CD228 knockdown enhanced the osteogenic differentiation of hBM-MSC

**Figure 4:**
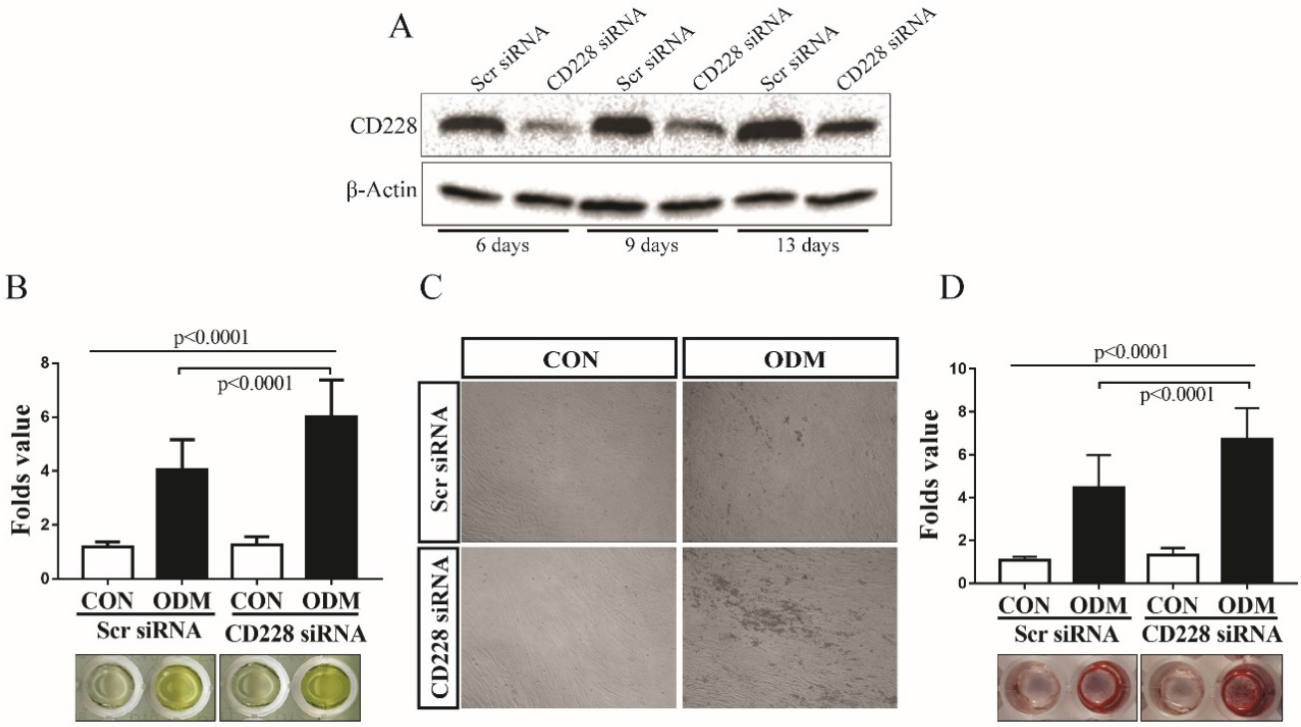
CD228 knockdown enhances osteogenic differentiation of hBM-MSC. (A) hBM-MSC were transfected with scrambled siRNA (Scr siRNA) or siRNA against CD228 (CD228 siRNA) for 48 h, collected by trypsinization, re-seeded, and kept for up to 13 days under osteogenic differentiation conditions. Western blot analysis for CD228 was performed at the indicated time points. β-Actin was used as an internal control. (B) Alkaline phosphatase activity was determined in Scr siRNA or CD228 siRNA-transfected hBM-MSC after the induction of osteogenic differentiation for 6 days. Alkaline phosphatase activity was normalized using MTT values for each group. Data is presented as the mean ± SD fold change of treated cells (ODM) compared to control (CON) cell of four independent experiments (n=5 for each). p-values were calculated using ANOVA or Student's t-test. (C) Phase contrast micrographs of Scr siRNA or CD228 siRNA-transfected hBM-MSC after the induction of osteogenic differentiation for 13 days. CON: control medium; ODM: osteogenic differentiation medium. Magnification: x 100. (D) Alizarin red S staining was performed to evaluate the mineralization of Scr siRNA- or CD228 siRNA-transfected hBM-MSC after 13 days of osteogenic differentiation induction. Alizarin red S staining intensity was normalized using MTT values for each group and expressed as the mean ± SD fold change of treated cells (ODM) compared to control (CON) cells for three independent experiments (n=5 for each). p-values were calculated using ANOVA or Student's t-test.

To determine the effect of CD228 knockdown on the osteogenic differentiation of hBM-MSC, we induced osteogenesis in both scrambled siRNA- and CD228 siRNA-transfected hBM-MSC for up to 13 days. We confirmed the effectiveness of the knockdown of CD228 by its specific siRNA for the duration of the differentiation period by western blot analysis (Figure 4A). We measured the activity of alkaline phosphatase (ALP) as an early signature of osteogenesis in hBM-MSC after 6 days of osteogenic differentiation induction. CD228 siRNA-transfected cells showed a higher level of ALP activity compared to control (Scr siRNA) in response to treatment with the osteogenic induction media (ODM) after 6 days (Figure 4B). Next, we analyzed the deposition of calcium by differentiated osteoblasts after 13 days of osteogenic differentiation induction of hBM-MSC by Alizarin red S staining. We observed higher mineralization in CD228-knocked down hBM-MSC evidenced by an increase in calcium deposition compared to control after 13 days of ODM treatment, which correlated with the higher ALP activity observed at an earlier stage of differentiation (Figure 4C and 4D). These findings confirmed that CD228 knockdown increased the osteogenic differentiation of hBM-MSC upon induction with ODM.

### CD228 knockdown enhanced the adipogenic differentiation of hBM-MSC

**Figure 5:**
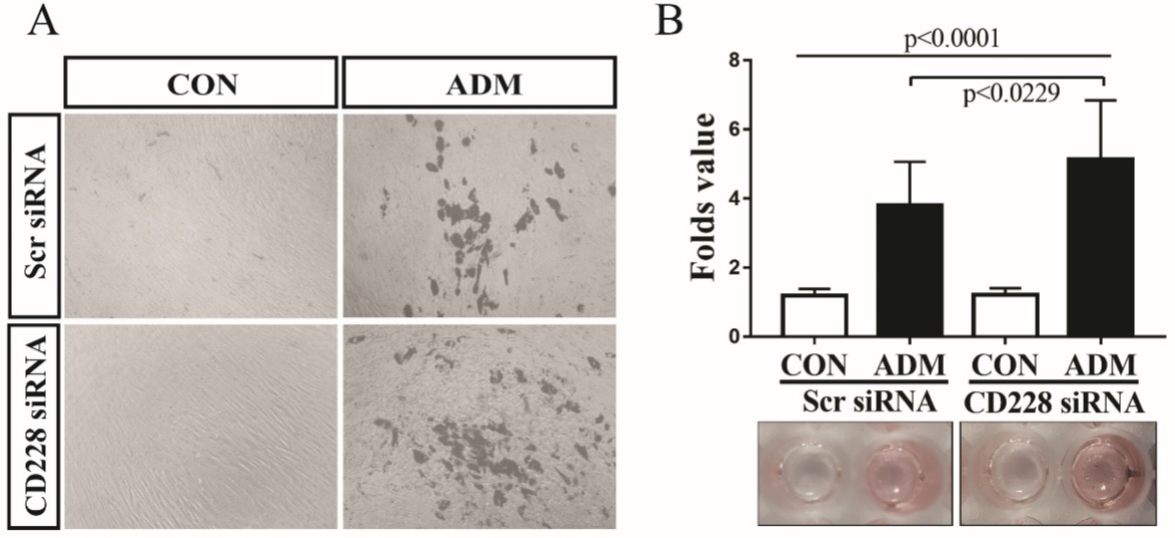
** CD228 knockdown enhances the adipogenic differentiation of hBM-MSC.** hBM-MSC were transfected with scrambled siRNA (Scr siRNA) or siRNA against CD228 (CD228 siRNA) for 48 h. Then, cells were collected, re-seeded, and kept for up to 16 days under adipogenic differentiation conditions. (A) Phase contrast micrographs of Scr siRNA or CD228 siRNA-transfected hBM-MSC after the induction of adipogenic differentiation for 16 days. CON: control media; ADM: adipogenic differentiation media. Magnification: x 100. (B) Differentiated adipocytes were stained with oil red O after 16 days to evaluate lipid accumulation. Oil red O staining intensity was normalized using MTT values for each group and expressed as the mean ± SD fold change of treated cells (ADM) compared to control cells (CON) for three independent experiments (n=5 for each). p-values were calculated using ANOVA or Student's t-test.

Accumulating information shows that commitment to either the osteogenic or the adipogenic lineage inhibits the differentiation towards the alternative lineage 58-60. We observed that CD228 knockdown increased the level of several markers and transcription factors which are essential for the osteogenic differentiation of hBM-MSC, but had no effect on adipogenesis-related genes (Figure 3B and 3C). Furthermore, CD228 knockdown increased the osteogenic differentiation potential of hBM-MSC (Figure 4). We then sought to evaluate whether CD228 knockdown would affect the adipogenic differentiation potential of hBM-MSC. We induced adipogenesis in scrambled- or CD228 siRNA-transfected hBM-MSC and evaluated the accumulation of lipids by oil red O staining after 16 days of treatment with adipogenic differentiation medium (ADM). Surprisingly, CD228 knockdown caused an increase in the adipogenic differentiation potential of hBM-MSC evidenced by a higher level of lipid accumulation compared to control (Figure 5A and 5B). These results suggest that CD228 might act as a suppressor for both osteogenic and adipogenic differentiation potential of hBM-MSC.

## Discussion

In this study, we identified CD228 as a cell surface marker that was expressed by young hBM-MSC, but not by old hBM-MSC or FB for the first time. Our results suggest that CD228 could potentially help in the identification and distinction of hBM-MSC from FB. We discovered that the differentiation potential of hBM-MSC towards the osteogenic and the adipogenic lineages increased in hBM-MSC when the expression of CD228 was downregulated by transfection of CD228-specific siRNA. These results suggest a role for CD228 as a modulator of the differentiation potential of hBM-MSC.

A wide array of research shows that FB can be easily mistaken for MSC, which necessitate definite criteria and procedures to distinguish between MSC and FB and avoid FB-contaminated MSC isolates 20-22, 24, 25, 61. It has been reported that MSC and FB share various cell surface markers that were originally proposed to identify MSC isolated from the bone marrow. Although these established positive markers, namely CD105, CD90, and CD73 do achieve the distinction between MSC and haematopoietic stem cells, they do not discriminate between MSC and FB. We found that these three surface markers were expressed in both hBM-MSC and FB, which supports the statements above (Figure 1). One of the solutions to this problem has been to look for specific markers to identify MSC. Several MSC-specific markers have been proposed, including CD106 and CD200 which are expressed by MSC, but not by FB. The specificity of such markers was further supported by our results showing that CD106 and CD200 were expressed by hBM-MSC but not by FB, allowing us to verify the identity of our hBM-MSC population (Figure 1). It is worth mentioning that although their presence and/or expression level vary according to the tissue of origin of MSC, the selection of MSC by use of these two cell surface markers, either alone or in combination with others, have been found to increase the CFU-F potential of MSC isolates. Furthermore, the expressions of both markers in MSC have been associated with their differentiation potential 30.

Here, we propose melanotransferrin (CD228) as a cell surface marker that is expressed in hBM-MSC but not in human FB, evidenced by RT-PCR, western blot analysis and FACS analysis (Figure 1). However, careful consideration should be taken to determine whether CD228 could act as a marker to identify MSC regardless of the tissue of origin (e.g. adipose tissue, umbilical cord, etc) or if it should be considered as a specific marker for MSC derived from the bone marrow as was found in other markers including CD200 62, 63. Naturally, other sources of FB should also be analyzed to confirm its absence in all FB sources, since FB can also show variations in the expression of cell surface markers depending on the tissues from which they are isolated 22, 64-66.

Knockdown experiments with CD228 siRNA transfection showed that the downregulation in the expression of CD228 upregulated the expression level of osteogenesis-related genes (RUNX2, DLX5, OC and Osx) and increased the osteogenic differentiation of hBM-MSC when cultured with osteogenic differentiation media (ODM), evidenced by an increase in ALP activity and Alizarin red S staining (Figure 3 and Figure 4). Despite there being no change in the expression level of PPARγ and aP2 in CD228 knocked-down hBM-MSC, the differentiation towards the adipogenic lineage also increased, as was evidenced by an increment in lipid accumulation under adipogenic differentiation conditions (Figure 3 and Figure 5). Enhanced osteogenesis and adipogenesis by knockdown of CD228 expression with CD228 siRNA, was further supported by our preliminary experiments using sorted hBM-MSC (CD228^high^ and CD228^low^), in which we observed higher osteogenic and adipogenic differentiation potential in CD228^low^ compared to CD228^high^ cells (data not shown). Overall, these results suggest that CD228 expression negatively regulates the differentiation of hBM-MSC towards both the osteogenic and the adipogenic lineages.

A study reported that CD228 was expressed in parallel with genes that regulate cartilage characteristics during differentiation 45. The same study showed that BMP-2, TGF-β, and insulin increased the expression of CD228 in the prechrondrogenic cells when these growth factors induced chondrogenic differentiation. CD228 was suggested to facilitate the differentiation of prechrondrogenic, although its overexpression alone was not sufficient to commit the mouse mesenchymal stem cell-like cell line C3H10T1/2 cells to the chondrocyte lineage 45. Therefore, it is possible that the expression of CD228 plays a positive role in regulating chondrogenesis of hBM-MSC, while suppressing osteo/adipogenic differentiation of them.

Silencing CD228 in hBM-MSC increased the expression of DLX5, RUNX2 and Osx all of which are transcription regulators required for the osteogenic differentiation of MSC. Especially, DLX5 is known to be a master regulator which can activate RUNX2 expression to promote osteogenesis, but inhibits adipogenic differentiation through PPARγ 67-69. Therefore, enhanced osteogenic potential might be ascribed to increased expression of DLX5 in the absence of CD228 in hBM-MSC. However, increased DLX5 expression by CD228 knockdown *per se* is not well reconciled with the promotion of adipogenic differentiation potential of hBM-MSC. Notably, DLX5 upregulation did not decrease the expression of PPARγ in CD228-silenced hBM-MSC as was reported previously, suggesting a novel mechanism that interferes DLX5-mediated suppression of PPARγ expression 69. The mechanism of increased adipogenic potential of CD228-silenced hBM-MSC in the presence of DLX5 upregulation as well as a mechanism of DLX5 upregulation upon CD228 knockdown remain to be elucidated by in-depth study.

Another notable finding in this study is a decrease in the expression of CD228 in older passages of hBM-MSC (Figure 2). A set of specific cell surface markers for the identification and isolation of MSC including CD106, CD146, and Stro-1 has been reported to be altered with aging of MSC 47, 70, 71. Variations in the multipotency of MSC such as loss of their multi-lineage differentiation potential are also observed as cells become senescent 47, 50, 72, 73. *In vitro* studies using MSC reported that aging negatively affects osteogenesis and less significantly, adipogenesis regardless of the culture conditions 72. On the contrary, aging tends to dysregulate the balance between adipogenesis and osteogenesis in the bone marrow by favoring the commitment/differentiation of hBM-MSC towards the adipogenic lineage at the cost of osteogenesis 74-76. Balance shift in differentiation potential accompanies with corresponding changes in gene expression 77, 78, which is in accordance with our findings that aging in hBM-MSC caused a decline in the expression level of two osteoblastogenic transcription factors RUNX2 and DLX5, and a significant increase in the expression level of two critical regulators of adipogenesis PPARγ and aP2 (Figure 2). In light of our data showing aging-associated decrease in the expression of CD228 and osteogenic transcription factors in hBM-MSC, we had assumed that CD228 could be involved in the upregulation of hBM-MSC differentiation towards the osteogenic lineage but inversely toward adipogenesis. On the contrary to our assumption, however, knockdown of CD228 expression did not inhibit specific differentiation lineage, but rather enhanced both adipogenic and osteogenic potential of hBM-MSC in this study. CD228 seems to act as a negative regulator for the differentiation of MSC towards these two lineages. Therefore, decreased CD228 expression with aging should be considered not directly associated with aging-dependent mutual exclusivity of adipogenic/osteogenic differentiation potential of hBM-MSC. Similar to CD228, loss of the expression of CD90 facilitated both osteogenesis and adipogenesis in MSC 79. It was suggested that knockdown of CD90 decreases stemness of MSC, promoting their differentiation when cultured in the appropriate conditions 79. Therefore, CD228 could be related with stemness of BM-MSC as it has been proposed for the cell surface marker CD90.

Overall, our data proposes CD228 as a marker that could be useful for distinguishing young hBM-MSC from old hBM-MSC and FB, and suggest a role for CD228 in the regulation of the differentiation potential of hBM-MSC towards the osteogenic and the adipogenic lineages. The exact mechanism of CD228 knockdown-associated enhancement of osteogenic/adipogenic differentiation potential remains to be elucidated by further studies.

## Materials and Methods

### Cell culture

Human BM-MSC were obtained from Sciencell Research Laboratories (Cat. No. 7500; Carlsbad, CA, USA) and were grown using α-Minimum Essential Medium (α-MEM; Gibco, Grand Islands, NY, USA) supplemented with 16.5% fetal bovine serum (FBS; SAFC Biosciences, Lenexa, KS, USA) and 1% penicillin and streptomycin (P/S, Gibco). Cells at passages 4-8 were used for experiments. In order to compare young and old hBM-MSC, passage numbers 4 to 6 were considered young, whereas passage numbers 16 to 18 were considered old. Human embryonic fibroblasts (FB) were cultured using Dulbecco's Modified Eagle's Medium (DMEM; Gibco) containing 10% FBS and 1% P/S 80. All cells were maintained at sub-confluency at 37 °C in a humidified incubator containing 5% CO2 and passaged using 0.05% Trypsin/EDTA (Welgene, Daegu, Korea).

### Small interfering (siRNA) transfection

Young hBM-MSC (passages 4-8) were seeded into 6-well plates at 1.5×10^5^ cells per well and cultured for 20-24 h using α-MEM supplemented with 10% FBS and without 1% P/S, prior to treatment with small interfering RNAs (siRNAs). Small interfering RNAs were transfected into cells at 40 nM using Lipofectamine^TM^ 2000 (Invitrogen, Carlsbad, CA, USA) following the manufacturer's instruction. After 48 h, cells were harvested for additional experiments. The sequences of the siRNAs (Genolution, Seoul, Korea) used in this study were as follow: negative control (scrambled) siRNA sense, 5'-CCUCGUGCCGUUCCAUCAGGUAGUU-3', negative control (scrambled) siRNA antisense, 5'-CUACCUGAUGGAACGGCACGAGGUU-3'; CD228 siRNA sense, 5'-GCGAUGUACUCAAAGCUGUUU-3', CD228 siRNA antisense, 5'-ACAGCUUUGAGUACAUCGCUU-3'.

### Differentiation of hBM-MSC

Young hBM-MSC (passages 4-8) were seeded at 8x10^3^ into 96-well plates using α-MEM supplemented with 10% FBS and 1% P/S. After 2-3 days of incubation, at 100% confluency, hBM-MSC were induced to differentiate. For osteogenic differentiation induction, confluent hBM-MSC were incubated for 13 days in osteogenic differentiation medium (ODM) consisting of α-MEM supplemented with 10% FBS, 100 nM dexamethasone (Sigma-Aldrich, St. Louis, MO, USA), 10 mM β-glycerophosphate (USB corporation, Cleveland, OH, USA), 50 μM ascorbic-2-phosphate (Sigma-Aldrich), and 1% P/S. Medium was changed twice per week. For adipogenic differentiation induction, confluent hBM-MSC were subjected to induction/maintenance cycles for 16 days. Each cycle consisted of incubating hBM-MSC for 3 days with adipogenic induction medium (ADM) composed of DMEM supplemented with 10% FBS, 1 μM dexamethasone, 0.5 mM 3-isobuty-l-methyl-xanthine (IBMX), 5 μg/ml insulin, 100 μM indomethacin (all above from Sigma Aldrich), and 1% P/S; followed by a 1-day incubation in adipogenic maintenance medium consisting of DMEM supplemented with 10% FBS, 5 μg/ml insulin, and 1% P/S.

### Alkaline phosphatase (ALP) activity assay

The activity of ALP was assessed as an early marker of osteogenic differentiation at day 6 after differentiation induction. Cells plated into 96-well plates were washed twice with PBS (phosphate buffered saline) and then fixed with 4% formaldehyde (Duksan, Gyeonggi-do, Korea) for 30 min at room temperature. ALP activity was determined colorimetrically by incubating cells with the substrate p-nitrophenyl phosphate (Sigma-Aldrich) diluted in a 1:15 ratio in alkaline buffer solution 1.5 M, pH 10.3 (Sigma-Aldrich) at 37 °C for 30 min. The absorbance was measured at 405 nm and normalized against each experimental group's MTT value with a Multiskan^TM^ GO microplate reader (Thermo Fisher Scientific, Waltham, MA, USA). Values were expressed as the fold change relative to control (undifferentiated) cells.

### Cell viability (MTT) assay

In order to assess cell viability and normalize the values obtained from the colorimetric measurement of the ALP assay, Alizarin S Red staining, and oil Red O staining, the viability of each experimental group was assessed at each corresponding time point by the methylthiazolyldiphenyl-tetrazolium bromide (MTT) assay 81. Briefly, cells plated into 96-well plates were incubated at specified conditions for an indicated time period. Then, after aspirating the culture medium, cells were incubated with 100 μl MTT solution (5 mg/ml MTT in PBS) at 37 °C. After 3 h, 100 μl of lysis buffer containing 10% SDS in 0.01N HCl were added into each well and, after overnight incubation, the absorbance at 562 nm was measured on the Multiskan^TM^ GO microplate reader.

### Alizarin red S staining

To evaluate the osteogenic differentiation of hBM-MSC, calcium deposition was assessed by staining differentiated cells with Alizarin red S. Briefly, cells were washed with PBS twice, fixed with 4% formaldehyde for 30 min at room temperature, rinsed with distilled water, and stained with 2% (w/v) Alizarin red S (Sigma-Aldrich) dissolved in distilled water (pH 4.2, adjusted with 10% ammonium hydroxide, Sigma-Aldrich) for 20 min. Then, cells were washed thoroughly with distilled water and examined for mineralization of the extracellular matrix (ECM). Following imaging, the dye was eluted using 10% (w/v) cetylpyridinium chloride monohydrate (Sigma-Aldrich) in 10 mM sodium phosphate (pH 7.0; Sigma-Aldrich) for 1 h at room temperature, and the absorbance was measured at 570 nm using the Multiskan™ GO microplate reader, and normalized against MTT values for each experimental group. Values were expressed as fold change relative to control (undifferentiated) cells.

### Oil red O staining

To evaluate the adipogenic differentiation of hBM-MSC, accumulation of lipid droplets in differentiated adipocytes was assessed by oil red O staining. Briefly, cells were washed with PBS twice and fixed with 4% formaldehyde for 30 min at room temperature. Then, cells were washed twice with PBS, incubated for 3 min with 60% isopropanol (Sigma-Aldrich), and stained with 0.6% oil red O (Sigma-Aldrich) in isopropanol for 2 h at room temperature. Cells were rinsed with distilled water 5 times and, after imaging, the dye was eluted with 100% isopropanol for 1 h. Absorbance was measured at 540 nm using the Multiskan™ GO microplate reader and normalized against MTT values for each experimental group. Values were expressed as fold change relative to control (undifferentiated) cells.

### Flow cytometry

To analyze the expression of cell surface markers, hBM-MSC or FB were typsinized, re-suspended in PBS containing 2% FBS, and stained with the following antibodies for 30 min at 4 °C: PE-conjugated anti-CD228 (R&D Systems, Minneapolis, MN, USA), APC-conjugated anti-CD106 (BioLegend, San Diego, CA, USA), PE-conjugated anti-CD90, PE-conjugated anti-CD73, APC-conjugated anti-CD105, PE-conjugated anti-CD200, PE-conjugated mouse IgG1 isotype control, or APC-conjugated mouse IgG1 isotype control (all from eBioscience, San Diego, CA, USA). Flow cytometry was performed on FACSCalibur^TM^ (BD Biosciences, Sparks, MD, USA) and analyzed with CellQuest Pro^TM^ software (BD Biosciences).

### RNA extraction and reverse transcription PCR (RT-PCR)

Total RNA was extracted using Tri-RNA (Favorgen, Ping-Tung, Taiwan) following the manufacturer's instruction. cDNA was synthesized using high capacity cDNA transcription kit (Thermo Fisher Scientific) according to the manufacturer's instruction. The PCR was performed as follows: one cycle of 3 min at 95 °C; 40 cycles of denaturation at 95 °C for 30 s, annealing at 57 °C for 30 s and extension at 72 °C for 45 s; followed by a final cycle of 5 min at 72 °C. The PCR products were loaded onto 1% agarose gel containing ethidium bromide (Promega, Madison, WI, USA). The expression of β-Actin mRNA level in each sample was analyzed as a control for input RNA amount. The primers that were used are listed in Table S1 of the Supplementary materials.

### Western blot analysis

In order to obtain total cell lysates, cultured cells were washed twice with ice-cold PBS and then lysed in RIPA lysis buffer including 50 mM Tris-HCl pH 7.4, 150 mM NaCl, 1 mM EDTA, 1% NP-40, 0.1% SDS (all from USB, Cleveland, OH, USA), 0.5% sodium deoxycholate (Sigma-Aldrich), 1 mM PMSF (Sigma-Aldrich), protease inhibitor cocktail (Pierce Biotechnology, Rockford, IL, USA), and phosphatase inhibitors containing 50 mM sodium fluoride, 2 mM sodium orthovanadate and 5 mM sodium pyrophosphate (all from Sigma-Aldrich). Protein concentration was determined using Bicinchoninic acid (BCA) assay (Thermo Fisher Scientific). Proteins were denatured by incubating at 95 °C for 10-15 min and equal amounts of total proteins (10-30 μg) were separated by SDS-PAGE. Proteins were then transferred onto Hybond-ECL nitrocellulose membranes (Amersham, Arlington Heights, IL, USA). The membranes were blocked with Tris-buffered saline Tween 20 (TBS-T: 10 mM Tris-HCl pH 7.6, 150 mM NaCl, and 0.1% Tween 20 (USB) containing 5% nonfat dry milk (Becton Dickson and Company, Sparks, MD, USA) or 5% bovine serum albumin (BSA; Amresco, Solon, OH, USA) for 1 h at room temperature and incubated overnight at 4 °C with specific primary antibodies. Primary antibodies against CD228 (1:500, Santa Cruz Biotechnology, Dallas, TX, USA), DLX5 (1:1,000, Abcam, Cambridge, UK), RUNX2 (1:2,000, Cell Signaling Technology, Beverly, MA, USA), PPARγ (1:1,000, Santa Cruz Bioteconology), and β-Actin (1:20,000, Sigma-Aldrich) were used for immunoblotting. The membranes were washed three times with TBS-T and then incubated with the appropriate horseradish peroxidase (HRP)-conjugated secondary antibodies for 1 h at room temperature. The blots were visualized using ECL detection reagents (Advansta, Menlo Park, CA, USA) and imaged using a ChemiDoc^TM^ MP Imaging system (BioRad, Hercules, CA, USA).

### Statistical analysis

Quantitative data are presented as the mean ± standard deviation (SD). The significance of experimental data was analyzed using ANOVA and unpaired two-tailed Student's t-test. Differences with p values of less than 0.05 were considered significant. Statistical analyses were performed using GraphPad version 7.00 (GraphPad Software, Inc. CA, USA; http://www.graphpad.com).

## Supplementary Material

Supplementary table S1.
