# Supplementary material for: The Role of Melanotransferrin (CD228) in the regulation of the differentiation of Human Bone Marrow-Derived Mesenchymal Stem Cells (hBM-MSC)
Source: Int J Med Sci. 2021 Feb 4;18(7):1580–91. doi: 10.7150/ijms.53650 (PMC7976559; doi:10.7150/ijms.53650)
Supplement: Supplementary file 1 — Supplementary table S1. [file ijmsv18p1580s1.pdf]

Supplementary Materials

Table S1. List of primers used in this study for RT-PCR analysis.

**Gene name**	**Forward/Reverse Primer (5′ to3″)**	**Size**
β-Actin	F-GGC ATC GTG ATG GAC TCC G<br>R-GCT GGA AGG TGG ACA GCG A	612 bp
DLX5	F-TTC CAA GCT CCG TTC CAG AC<br>R-GTA ATG CGG CCA GCT GAA AG	408 bp
Osteocalcin (OC)	F-GAG CCC TCA CAC TCC TC<br>R-CCG TAG AAG CGC CGA TAG	228 bp
aP2	F-AAC CTT AGA TGG GGG TGT CCT G<br>R-TCG TGG AAG TGA CGC CTT TC	124 bp
CD228	F-GAT GGG CTG CGA TGT ACT CA<br>R-CAT CGT CCT ACG TGC TTC CT	334 bp
Osterix (Osx)	F-CCA CCT ACC CAT CTG ACT<br>R-GTT TGG CTC CAC CAC TCC	155 bp
RUNX2	F-ACT GGG CCC TTT TTC AGA<br>R- GCG GAA GCA TTC TGG AA	316 bp
PPARγ	F-GCT GTG CAG GAG ATC ACA GA<br>R-GGG CTC CAT AAA GTC ACC AA	205 bp
